# The Adoption of the GameSquad Exergaming Intervention for Young Adults with Down Syndrome: A Qualitative Analysis

**DOI:** 10.18103/mra.v13i6.6608

**Published:** 2025-06

**Authors:** Kameron Suire, Brian Helsel, April Bowling, Amanda E. Staiano, Joseph Sherman, Annie Rice, Lauren Ptomey

**Affiliations:** 1 Berry College; 2 University of Kansas Medical Center,; 3 Merrimack College; 4 Pennington Biomedical Research Center

## Abstract

**Introduction:**

Adults with Down syndrome face persistent barriers to physical activity, including mobility limitations, lack of accessible programming, and low self-efficacy. Exergaming represents a potentially scalable, home-based approach to increasing physical activity in this population.

**Purpose:**

This qualitative study explored the experiences, preferences, and perceived barriers of young adults with Down syndrome and their live-in caregivers who participated in a 12-week exergaming intervention. The program paired the narrative-based game *Ring Fit Adventure*^™^ (Nintendo Switch^™^) with weekly 15-minute virtual health coaching sessions designed to review weekly progress, reinforce the importance of moderate-to-vigorous physical activity address any technical issues (e.g., related to the game, system, or Fitbit), and provide social support and encouragement.

**Methods:**

Twenty adults with Down syndrome (*M* age= 23.2 ± 3.9, 85% non-Hispanic white, 65% female) enrolled and 19 completed the trial. One participant ceased communication with the research team after completing pre-testing. Semi-structured exit interviews were conducted with participants and their caregivers. Interviews were transcribed verbatim and analyzed using reflexive thematic analysis. Themes were developed iteratively using a six-step framework.

**Results:**

Through analysis, we identified five distinct themes that reflect shared and unique experiences related to 1) engagement, 2) autonomy, 3) physical challenge, 4) technology, and 5) long-term value. Participants were motivated by the immersive game design and reported a preference for playing independently. Caregivers described their roles in supporting routine formation and providing occasional technical assistance.

**Conclusion:**

Exergaming, supported by light-touch health coaching, was perceived as an engaging, accessible, and sustainable strategy to increase physical activity among young adults with Down syndrome. These findings support the design of home-based interventions that promote autonomy and long-term adherence for individuals with intellectual disabilities.

## Introduction

Down syndrome (DS), or trisomy 21, is the most common chromosomal cause of intellectual disability, affecting approximately 1 in 714 live births in the United States.^1^ Advances in healthcare, early intervention, and supportive services have led to a dramatic increase in life expectancy for individuals with DS, with many now living into their 50s and 60s.^2,3^ As this population ages, however, they face distinct and premature declines in functional health, including reduced mobility, balance issues, frailty, musculoskeletal complications, and elevated rates of obesity and chronic conditions.^4–6^ These challenges are compounded by low levels of physical activity (PA), which further accelerate declines in physical function.^7^ Despite well-established benefits of moderate-to-vigorous physical activity (MVPA) for improving strength, mobility, and overall function, fewer than 10% of adults with intellectual disabilities, including those with DS, meet recommended PA guidelines.^8^

Barriers to PA engagement for adults with DS are well-documented. These include biomechanical challenges such as gait abnormalities,^9^ limited transportation access, lack of modified fitness environments, low self-confidence in typical exercise settings, and limited social support.^10,11^ Critically, the transition period from adolescence to adulthood (ages 18–25) often brings added disruption to routines, medical support, and structured daytime activities, contributing to further decreases in PA during a stage when independence is rising but supports often fall away.^12,13^ Effective interventions for this age group must therefore be not only physically appropriate but also socially and cognitively accessible, acceptable to the home environment, and intrinsically motivating.

Exergaming, video games that require physical movement to play, has emerged as a promising, low-barrier approach to increasing PA in a range of populations, including individuals with Intellectual disabilities.^14–16^ Exergames may offer a particularly useful format for young adults with DS by combining structured movement, visual and auditory feedback, and playful, goal-driven environments. Research in children and adolescents suggests that exergaming can improve cardiovascular fitness, muscular strength, and functional mobility while increasing motivation and enjoyment.^17–18^ However, less is known about how adults with DS perceive and experience exergaming, especially in real-world settings where sustainability, autonomy, and ease of use are critical to long-term engagement.

To our knowledge, no studies have qualitatively explored the preferences, barriers, and experiences of young adults with DS using exergaming as a strategy to improve physical activity and function. The recently completed 12-week GameSquad-DS intervention offered a unique opportunity to fill this gap. The program paired a home-based exergame, Ring Fit Adventure^™^ (Nintendo Switch^™^), with weekly virtual health coaching designed to encourage use and troubleshoot any technology or motivational issues. While quantitative outcomes have been reported elsewhere,^19^ this study presents a qualitative evaluation of participant and caregiver perspectives.

The aim of this study was to better understand how young adults with DS and their caregivers experienced the GameSquad-DS intervention, including what aspects supported or hindered participation. Through thematic analysis of exit interviews, this study sought to explore key elements of engagement, autonomy, physical challenge, technological usability, and sustainability. Findings from this work may help inform the refinement of accessible, enjoyable, and empowering PA interventions that support long-term physical function and well-being in adults with DS.

## Methods

This qualitative analysis draws data from semi-structured interviews with young adults with DS and their parental caregivers who participated in a 12-week feasibility trial of the GameSquad-DS intervention. Details of the intervention and primary outcomes have been previously published.^19^ It is important to mention this study’s design took inspiration from a previous home-based exergaming intervention.^20^ Briefly, participants were asked to engage with the game for a minimum of 120 minutes per week over 12 weeks and attend weekly 15-minute virtual health coaching sessions with their caregiver. These sessions were designed to review weekly progress, reinforce the importance of MVPA, address any technical issues (e.g., related to the game, system, or Fitbit), and provide social support and encouragement. Gameplay goals increased incrementally over the intervention period: 60 minutes in week 1, 80 minutes in week 2, 100 minutes in week 3, and 120 minutes during weeks 4–12. Participants began at the first level of the game and progressed through multiple levels and worlds, each comprising five to ten stages that typically lasted 10–15 minutes. To advance through levels and battle in-game “monsters,” participants performed various exercises using the Pilates-style ring and leg strap (e.g., squats, overhead presses, and bow pulls). Successful completion of levels and battles unlocked new exercises, outfits, and other in-game rewards to reinforce engagement and progression.

Individuals were interviewed at the conclusion of the trial to examine barriers to participation, positives, and negatives relative to both the exergame and health coaching components of the intervention, their overall experience with the program, and suggestions for modifications to the health coaching sessions. These interviews took place over Zoom^™^ video conferencing or in person at a Midwestern academic medical center in January 2023 to June 2023. Informed consent was obtained by all participants and procedures were approved by the University’s Institutional Review Board. All interviews were conducted by the first author.

To better understand caregivers’ perspectives on the feasibility, effectiveness, and overall experience of the intervention, we asked a series of open-ended questions. These questions were designed to elicit insights on gameplay observations, perceptions of long-term use, levels of involvement, and emotional responses to supporting the intervention. This information helped us explore both the direct and indirect impacts of the program on family dynamics, support needs, and perceived value. The interview guide consisted of five questions directed to the caregiver of the adult with DS, and these questions are presented in Table 1.

To capture the voices of young adults with Down syndrome and understand their direct experiences with the intervention, we posed a set of accessible questions. These were aimed at exploring participants’ enjoyment, challenges, preferences, and willingness to continue using the game independently. Their responses offered critical insight into motivation, engagement, and personal ownership of the program. Four questions were asked directly to the young adult with DS and are presented in Table 2.

All interviews were audio-recorded and manually transcribed verbatim by the lead author immediately following each interview to preserve detail and contextual accuracy. Transcripts were anonymized during transcription to maintain confidentiality. Data were analyzed using reflexive thematic analysis, guided by the six-phase framework outlined by Kiger and Varpio^21^ and based on Braun and Clarke’s foundational methodology.^22^

This process involved (1) familiarization with the data through repeated reading, (2) generation of initial codes across the dataset, (3) construction of potential themes by clustering related codes, (4) review and refinement of themes for internal coherence and external distinction, (5) defining and naming finalized themes, and (6) producing the report with illustrative quotes. The lead author conducted all coding and theme development using a recursive, iterative approach. Although a single-coder strategy was employed, methodological rigor was enhanced through ongoing memoing, maintenance of a coding audit trail, and reflexive note-taking to support transparency and consistency.

## Results

Nineteen participants completed the full 12-week intervention. One participant withdrew after pre-testing and ceased communication with the research team. Nineteen interviews were analyzed. Interview length ranged from approximately 15 to 35 minutes, with a mean duration of 26 minutes (SD = 4.8). Basic sociodemographic data on caregivers and adults with DS are described in Table 3.

Through analysis, we identified five distinct themes: reflected shared and distinct experiences related to engagement, autonomy, challenge, technology, and long-term value. The themes are presented below in Table 4, followed by narrative summaries and illustrative quotes.

## Engagement Through Gamification and Narrative

Participants and caregivers frequently described the game’s storyline, characters, and level progression as key motivators. The structure transformed exercise into an immersive experience, promoting consistent engagement and a sense of accomplishment. This narrative-driven design helped participants stay focused and made exercise feel purposeful rather than repetitive. The game’s reward system and character progression gave them something to look forward to and return for regularly.

- Participant: “I liked the exercise parts. I also liked getting past all of the guys.”
- Caregiver: “He got attached to the game and the characters. That made him want to come back.”
- Participant: “I like fighting the monsters and moving to the next world.”

## Autonomy and Ownership

Many participants preferred to engage with the game on their own terms, often in a private space. Caregivers supported this independence, noting that participants were more motivated when given control over how and when they played. For several caregivers, this independence marked a meaningful step toward self-determination, a developmental goal often difficult to support in traditional physical activity settings. The minimal need for supervision enhanced participants’ confidence in navigating the program.

- Caregiver: “She wanted to do it on her own… It wasn’t as fun for her when I joined.”
- Participant: “I did it on my own. In the basement, usually.”
- Caregiver: “He didn’t want me playing with him. He felt like it was his thing.”

## Physical Demands

Participants and caregivers described the physical demands of the game with a mix of challenge and enjoyment. Movements like squatting, running in place, and engaging in battles were often labeled as “hard,” but rather than deterring use, these physical challenges appeared to enhance motivation and provide a sense of progress. Participants frequently described difficult gameplay with pride and perseverance, indicating that physical exertion contributed to a feeling of mastery and accomplishment. Caregivers echoed these observations, noting that the physical challenge embedded within the game often pushed participants further than traditional exercise. Many reported that their sons or daughters were more willing to engage in exercise through the game than through typical outdoor activities or structured workouts.

- Caregiver: “Some of it was hard for him, but he didn’t quit. He kept going because he wanted to level up.”
- Participant: “It was hard, but I kept trying because I wanted to beat the level.”
- Caregiver: “She was doing more exercise than I expected. She doesn’t like walking outside, but she loved this.”

## Technological Usability and Barriers

Most technical difficulties were minor, such as slipping leg straps or syncing devices. Participants rarely mentioned these problems, suggesting that caregivers’ troubleshooting or health coaching support helped minimize disruption. In some cases, initial setup required assistance, but once routines were established, participants often managed independently. Virtual coaching served as a safety net, reducing dropout risk from technical frustrations.

- *Caregiver, RF006:* “Only frustration was syncing the Fitbit.”
- *Participant, RF013:* “I needed help with the leg strap one time.”
- *Caregiver, RF009:* “The controller slipping down… that’s it.”

## Sustainability and Future Use

Participants frequently expressed enthusiasm for continuing with the game post-study. Caregivers also recognized its potential as a lasting physical activity option, especially due to its convenience and flexibility. Some noted that it was especially useful during inclement weather or when outdoor options were limited. The appeal of gaming, combined with embedded movement, allowed caregivers to view the program as both enjoyable and functional.

- *Participant, RF007:* “That is my first choice! I want to keep it.”
- *Caregiver, RF026:* “We’re going to keep using it. It’s a routine now.”
- *Caregiver, RF015:* “I’m excited to have it for the winter months.”

## Discussion

The results of this qualitative analysis align with and extend the existing literature on physical activity promotion among individuals with Down syndrome. Participants and caregivers reported high levels of engagement, motivation, and autonomy, consistent with prior work emphasizing the importance of intrinsic motivation and perceived competence in sustaining behavior change.^23–25^ These findings echo literature showing that when individuals with intellectual disabilities enjoy and feel ownership over an activity, adherence improves.^26,27^·

The emphasis on autonomy, particularly participants’ desire to engage with the intervention independently, supports findings that self-directed activity enhances self-efficacy and motivation in individuals with intellectual disabilities.^28,29^ Caregiver accounts of “stepping back” to allow independence reflect principles of supported self-management, a model of care promoting autonomy through light-touch guidance.^30^ This also aligns with Self-Determination Theory, which emphasizes autonomy as a core psychological need that supports motivation and long-term behavior change.^23^ In addition to autonomy, participants appeared to develop a sense of competence through progressive gameplay challenges and visual feedback systems. The weekly virtual coaching sessions and caregiver encouragement may have supported relatedness, reinforcing the social aspect of motivation outlined in SDT.^31^ Together, these elements suggest that well-designed digital interventions can effectively meet all three SDT needs, supporting internal motivation and continued engagement.

The findings also have important implications for the design and scalability of future physical activity interventions targeting individuals with intellectual disabilities. The success of this program hinged not only on its narrative and physical challenge elements but also on its flexibility, accessibility, and minimal training requirements. Developers and clinicians designing future interventions can draw from these features to maximize engagement: creating game-based platforms that incorporate adaptive difficulty, immersive storytelling, and clear rewards systems while minimizing technical barriers.^16,20,21^ This is especially relevant given prior work by Shields et al., who found that a community-based progressive resistance training program improved physical function in adults with Down syndrome without compromising adherence or enthusiasm.^32^ These findings reinforce the idea that appropriately scaffolded physical challenge, whether in traditional or gamified formats, can enhance motivation and self-efficacy in this population. Additionally, the integration of brief, virtual coaching allowed for scalable support without demanding significant caregiver or clinical time, addressing a known bottleneck in program implementation.^16,33^ These insights underscore the value of pairing digital interventions with light-touch human connection, especially for individuals who may face social or cognitive barriers to traditional exercise programming.^32,34^ These design principles may also apply to other health promotion areas, such as diet, sleep, or stress management, when integrated with behavioral feedback and goal-setting systems.^35^ Moreover, the approach may be adaptable for other populations with cognitive or developmental disabilities, expanding its potential reach and utility.^36^

Caregiver perspectives in this study revealed both the promise and complexity of implementing home-based digital interventions within the context of intellectual disability. While caregivers largely reported low levels of burden and occasional technology troubleshooting, the broader issue of digital access and equity remains a consideration. Not all families may have reliable internet access, access to gaming consoles, or the technological literacy needed to support setup and maintenance.^32,37^ It is also important to consider that families who chose not to participate may have faced greater technological or logistical barriers, potentially introducing selection bias. Future efforts should consider strategies such as device lending programs, partnerships with local disability service organizations, or community-based tech navigators to expand access and reduce inequities.^38^ Nevertheless, the dual-perspective approach employed in this study, capturing both caregiver and participant voices, strengthens the ecological validity of the findings.^26,27^ The home-based, low-barrier design and high completion rate (19 of 20 participants) further emphasize the feasibility and acceptability of this model in real-world settings.^19^ As the field continues to prioritize inclusion and sustainability in intervention design, programs like GameSquad-DS offer a promising blueprint for how to align behavior change theory with everyday usability.

This study offers several unique strengths that enhance its contribution to the literature. First, the inclusion of both participant and caregiver perspectives allowed for a more comprehensive understanding of engagement, barriers, and motivation within the home environment. Second, the intervention was tested under real-world conditions, with minimal in-person contact or external prompting, increasing its ecological validity. The high completion rate (95%) and consistent engagement across the 12-week period demonstrate the feasibility of this approach in a community setting. Finally, the focus on young adults with Down syndrome, a population underrepresented in physical activity research, addresses a critical gap and highlights the importance of tailored, inclusive intervention design.

## Limitations

This study has several limitations. The generalizations are limited by a small sample (n= 20) of young adults with DS, a short-term study (12-week), and non-randomized trial, and thus should be interpreted cautiously. Future interventions should consider increasing the sample size, introducing a control group, and increasing the duration of the intervention. The sample also may reflect families who were more engaged, resource-supported, or technologically equipped, given that recruitment relied on self-referral through community outreach and the use of video chat for eligibility screening. This may limit the generalizability of findings to individuals with lower levels of caregiver involvement, limited access to technology, or those who receive services outside of community-based organizations. Interviews were also conducted with the participants and caregivers together and it is possible this may have influenced answers in some manner. Finally, all interviews were transcribed and analyzed by a single researcher. While this approach enabled consistency in interpretation, it may have limited the diversity of analytical perspectives.

## Conclusions

This study demonstrates the potential of narrative-driven exergaming, paired with brief health coaching, to support physical activity engagement among young adults with DS. Through both participant and caregiver perspectives, this exergaming intervention was shown to promote enjoyment, autonomy, physical challenge, and sustainability, factors that are critical yet often underemphasized in intervention design for this population.

By addressing common barriers such as transportation, motivation, and accessibility, the adopted GameSquad-DS intervention offers a scalable, home-based strategy to increase MVPA. These findings support the integration of gamified, user-centered technologies into future public health and clinical efforts aimed at improving physical function and lifelong wellness in individuals with intellectual disabilities.

## Figures and Tables

**Table 1:** Caregiver Interview Questions

Caregiver Interview Questions
What are your thoughts overall on how the game went from your observations/experience? Do you think the game could be a continued option for physical activity for your child? Why or why not? How involved did you feel you needed to be for your child during the study? (Helping them with the game, encouragement, etc.) How did you feel when you were involved with study requirements? (Coaching sessions, helping your child, etc.) What other thoughts do you have about your experience that could be useful for us?

**Table 2:** Participant Interview Questions

Participant Interview Questions
How would you describe your experience using the game? What did you like about the game? What didn’t you like about the game? Will you continue using the game after this study?

**Table 3:** Demographic Data on Young Adults with DS

Demographics	Overall	Male	Female
	N = 20	N = 7	N = 13
Age	23.5 ± 3.9	23.1 ± 4.1	23.7 ± 4.0
**Ethnicity**			
Hispanic or Latino	2 (10 %)	0 (0 %)	2 (15 %)
Not Hispanic or Latino	18 (90 %)	7 (100 %)	11 (85 %)
**Race**			
Native Hawaiian or Pacific Islander	1 (5.0 %)	1 (14 %)	0 (0 %)
Black or African American	1 (5.0 %)	1 (14 %)	0 (0 %)
White	18 (90 %)	5 (71 %)	13 (100 %)

**Table 4.** Overarching Themes Identified in Participant and Caregiver Interviews

Theme	Description
*Engagement Through Gamification and Narrative*	Participants were motivated by the storyline, battles, and progression structure of the game.
*Autonomy and Ownership*	Participants preferred independent gameplay, supported by caregivers’ encouragement.
*Physical Demands*	While some activities were difficult, they often motivated rather than deterred engagement.
*Technological Usability and Barriers*	Technical issues were minor and mostly manageable with caregiver or coach support.
*Sustainability and Future Use*	Participants expressed interest in continuing the game after the study’s conclusion.
